# Transcriptomic analysis of differential expression between surviving and nonsurviving patients infected by the SARS-CoV-2 Delta variant

**DOI:** 10.1038/s41598-025-00280-3

**Published:** 2025-05-15

**Authors:** Ivan Vlasov, Tatiana Usenko, Alexandra Panteleeva, Mikhail Nikolaev, Artem Izumchenko, Valeriia Panafidina, Elena Gavrilova, Irina Shlyk, Valentina Miroshnikova, Yurii Polushin, Maria Shadrina, Sofya Pchelina, Petr Slominsky

**Affiliations:** 1 National Research Center «Kurchatov Institute», Moscow, Russian Federation; 2 Pavlov First Saint-Petersburg State Medical University, Saint-Petersburg, Russian Federation; 3 Petersburg Nuclear Physics Institute named by B.P. Konstantinov of National Research Center «Kurchatov Institute», Saint-Petersburg, Russian Federation; 4 Kurchatov Genome Center—PNPI, Saint-Petersburg, Russian Federation

**Keywords:** Peripheral blood mononuclear cells, COVID-19, Delta SARS-CoV-2, Transcriptome, Gene ontology, Transcriptomics, Molecular medicine, Infectious diseases, Respiratory tract diseases

## Abstract

For a more precise understanding of the course of the pathological process in patients with severe COVID-19, it is necessary to continue the search for factors that affect the course of the pathological process and the possibility of a favorable outcome in critically ill patients. Comparative RNA-seq analysis of the transcriptome of peripheral blood mononuclear cell (PBMCs) in patients with a severe clinical course of COVID-19 caused by the SARS-CoV-2 Delta strain revealed a number of differentially expressed genes that distinguish patients with different clinical outcomes (survivors vs. nonsurvivors) in the period of 30 days after admission to the hospital. Most of them are associated with the “negative regulation of viral process” and “negative regulation of immune response” clusters. Moreover, in surviving patients, there is increased expression of the key genes *C1QB*, *C1QA*, *ISG15*, *SERPING1*, *VSIG4*, *KLRD1*, *TRPM4*, and *HFE* incorporated in these clusters. Among these key genes, the *ISG15* gene, which links several clusters of gene ontology enrichments and encodes an interferon-induced ubiquitin-like protein, deserves special attention. Its product, ISG15, is known to be a primary substrate for SARS-CoV-2 protease PLpro, which plays a role in counteracting hosts’ antiviral mechanisms.

**Supplementary Information:**

The online version contains supplementary material available at 10.1038/s41598-025-00280-3.

## Introduction

In December of 2019, the first patients with a novel form of pneumonia caused by infection with SARS-CoV-2 virus were described in the Wuhan province of China. Previously, two other pneumonia-inducing coronaviruses—MERS-CoV and SARS-CoV—had been described. All three of the aforementioned coronaviruses cause a severe form of pneumonia (with an up to 36% lethality rate in the case of MERS-CoV); however, SARS-CoV-2 is the most transmissible^1^. Its high transmissibility and quick spread led to a global pandemic, resulting in more than 700 million cases and more than 7 million deaths worldwide^2^.

RNA viruses, and SARS-CoV-2 in particular, are characterized by high genome instability, which is caused by errors in replication of viral RNA^3^. From the standpoint of SARS-CoV-2 infection pathophysiology, mutations in spike glycoprotein S are especially interesting, since this is the protein that allows SARS-CoV-2 to invade host cells via binding to angiotensin-converting enzyme 2 (ACE2)^4^. Overall, since 2019, more than 400 mutations have been identified in S glycoprotein^5–9^. However, only some of those noticeably affected virus properties. Such impactful mutations have caused several SARS-CoV-2 variants to emerge, causing several waves of pandemic, each wave dominated by its own virus variant.

The first variant to emerge was the B1.1.7 variant, also known as the Alpha variant. It was initially identified in Great Britain in September of 2020 and later spread to more than 200 countries all over the world^10^. This variant has 23 mutations differentiating it from the original Wuhan variant, three of which—N501Y, D614G, and P681H—are considered to be most impactful in causing an increase in the transmissivity and severity of the virus. After that, several other impactful variants emerged, including Beta (B.1.351, identified in October 2020 in South Africa), Delta (В.1.617.2, identified in December 2020 in India), Gamma (В.1.1.28, identified in January 2021)^10^and Omicron (В.1.1.529, identified in November 2021 in South Africa). Currently, the Omicron variant and its subvariants dominate the landscape of SARS-CoV-2 genetic variability^11^.

Mutations in variants of the virus (both in S glycoprotein and other genes) can affect the properties of the virus on both clinical and molecular levels. When compared to previously emerged variants, the Delta variant of SARS-CoV-2 carries more than ten novel mutations in spike protein^12,13^, including two major mutations in receptor binding domain^13^. These mutations are posited to lead to increased infectivity, severity, and immune avoidance of this variant^12^, as well as stronger affinity to ACE2^13^.

Previously, we have shown that, in peripheral blood monocytes (PMBCs) of patients with severe COVID-19 caused by the Alpha variant of SARS-CoV-2, expression of genes related to low-density lipoprotein (LDL), the metabolic pathway was increased in patients with lethal outcomes^14^. As virus continues to evolve, it is necessary to re-examine previous findings and establish, if mechanisms that previously were found to be important in determination of the outcome still play the same role in COVID-19 cases, caused by the infection with new strains of virus. In this study, our goal was to replicate the design of our previous study while investigating a different SARS-CoV-2 variant. For that reason, we have investigated differential expression in primary blood monocytes between SARS-CoV-2 survivors and nonsurvivors, with both groups being confirmed to be infected by the Delta coronavirus variant. As in the previous paper, we have compared intensive care unit (ICU) patients with lethal and nonlethal outcomes at 30 days since hospitalization.

## Materials and methods

### Patients

From June 25, 2021, to August 12, 2021, 66 patients with severe pneumonia were admitted to the ICU of Pavlov First State Medical University of St. Petersburg. Patients were filtered based on the following inclusion criteria: Delta SARS-CoV-2 positive COVID-19 diagnosis as confirmed by RT-PCR, Russian ethnicity, age between 50 and 70 years, absence of chronic comorbidities (cancer, cerebrovascular diseases, heart failure, renal failure). Twenty patients met the aforementioned inclusion criteria. The situation of the patients was monitored for 30 days, and with ten patients dying and ten patients surviving in this period. Ten (five + five) patients from these groups were enrolled in the present study. The baseline demographic and clinical characteristics of the patients are summarized in Table 1. In all cases, the sample was collected at the moment of hospitalization to the ICU, the outcome was recorded 30 days after hospitalization, and RNA was extracted 7–21 h after sample collection.

The recruitment for this study was conducted from June 2021 until September 2021. The study was conducted in accordance with the World Medical Assembly Declaration of Helsinki: Ethical Principles for Medical Research Involving Human Subjects. All blood samples were collected with the informed consent of the investigated patients. The study was approved by the Ethics Committee of Pavlov First State Medical University of St. Petersburg (Russia). All patients provided their written informed consent for participation in this study.

**Table 1:** Clinical characteristics and RNA integrity number (RIN) of participating individuals.

Survival Status	Age	RIN	Body Temperature at Hospitalization	Oxygen Saturation	Respiration Rate	Ground Glasses’ Opacity Severity Based on CT-SKAN (quartile)	Day of Death/Discharge
Deceased	61	8.2	36.8	70	23	3	4
Survived	60	9.3	37.8	75	24	3	10
Survived	45	9.2	37.2	86	24	3	16
Deceased	48	9.6	36.9	90	24	4	9
Survived	63	8.6	37.5	90	16	4	34
Deceased	43	9.1	36.7	89	22	3	9
Deceased	73	9.3	36.7	89	19	3	12
Survived	43	7.6	38.6	90	17	2	17
Survived	58	9.1	37.1	94	17	2	10
Deceased	66	8.8	36.70	97	18	2	32
Deceased	45	9.7	37.3	97	16	2	26
Survived	59	8.1	37.1	75	30	2	18

### RNA isolation, library preparation, and sequencing

Peripheral blood mononuclear cells (PBMCs) were isolated from 8 mL of EDTA anticoagulated venous blood by a Ficoll-Paque gradient method (Ficoll-Paque PLUS, GE Healthcare, Chicago, IL, USA). After centrifugation, PBMCs were collected from the interface and washed twice with PBS (pH 7.4) to remove the platelet-rich plasma fraction. The PBMC cell pellets were aliquoted and resuspended in TRIzol Reagent (Thermo Fisher Scientific, Waltham, MA, USA) and immediately frozen at 80^o^C.

Total RNA was extracted from PBMCs of SARS-COV-2 infected patients using TRIzolTM Reagent (Thermo Fisher Scientific, USA) following the manufacturer’s protocol. All RNA samples were stored at 80^o^C for subsequent analysis. RNA quality and quantity were analyzed using a NanoDrop spectrophotometer system (Thermo Fisher Scientific) and an Agilent 2100 Bioanalyzer (Agilent Technologies, Santa Clara, CA, USA) with the RNA 6000 Nano Kit according to the manufacturer’s instructions. All samples had an RNA integrity number (RIN) > 8. Then, total RNA was used to obtain polyA fractions using oligoT magnetic beads Dynabeads mRNA Purification Kit (Ambion, Austin, TX, USA) according to the manufacturer’s protocol. Further, libraries for massive parallel sequencing were prepared from polyA RNA using a NEBNext Ultra II RNA Library Prep Kit (NEB, Ipswich, MA, USA) and NEBNext Multiplex Oligos for Illumina (Index Primers Set 1) according to the kit instructions. The concentration of the libraries was determined using Qubit dsDNA HS Assay Kit with a Qubit 2.0 Fluorometer (Thermo Fisher Scientific). The lengths of distribution fragments from the cDNA libraries were assessed on an Agilent 2100 Bioanalyzer (Agilent Technologies) using the Agilent High Sensitivity DNA Kit (Agilent Technologies). Unpaired (50-bp reads). Sequencing of these libraries was performed on the Illumina HiSeq1500 platform using the TruSeq SBS Kit V3 sequencing reagents (Illumina, San Diego, CA, USA). RNA-Seq data for each sample (raw and processed data) can be accessed in the Gene Expression Omnibus, accession no. GSE272392.

### Data processing and analysis

**Fig. 1:**
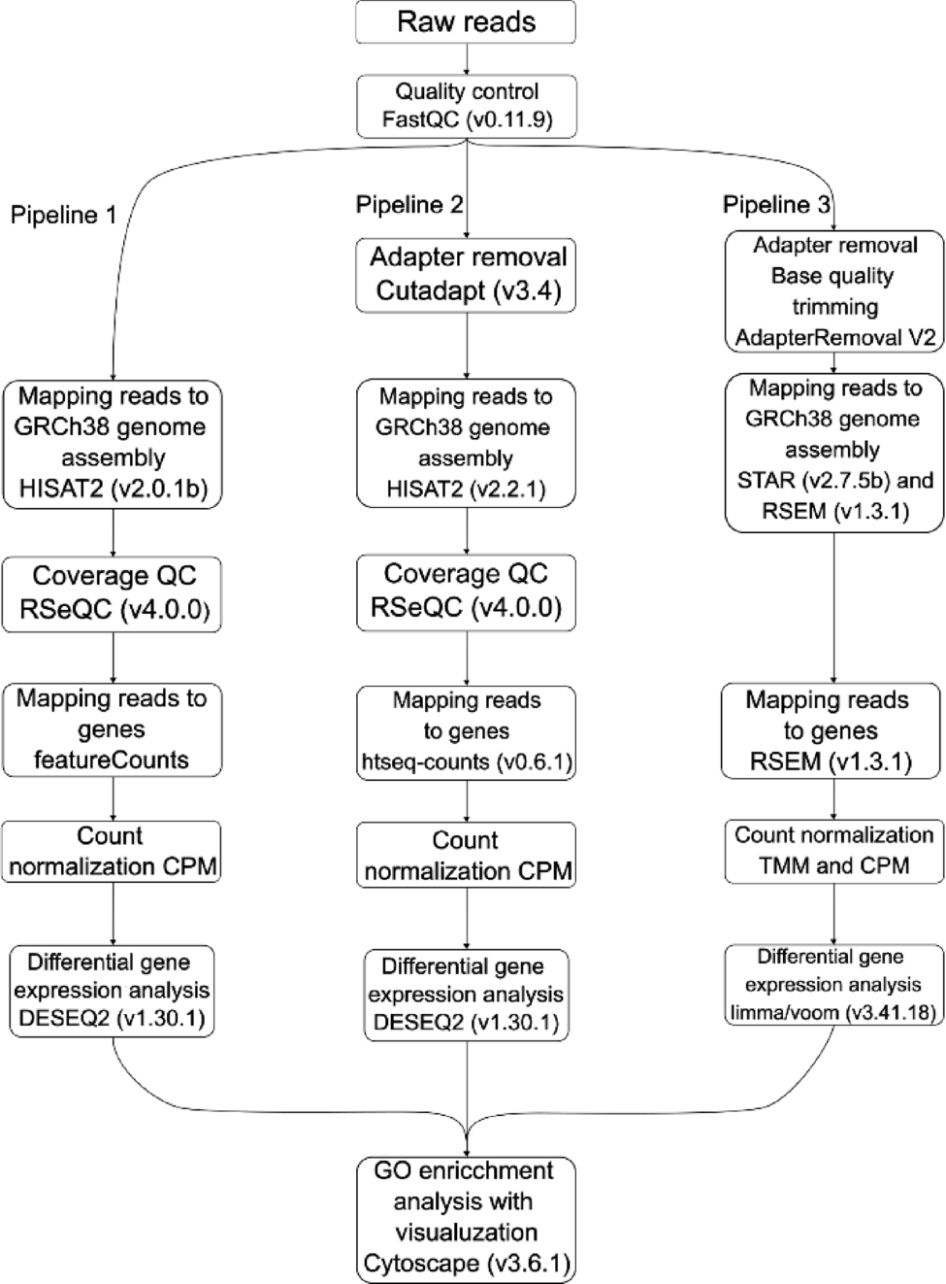
Description of pipelines used during data processing.

RNA-seq data were analyzed to identify differentially expressed genes (DEGs) using three pipelines according to the block diagram in Fig. 1. Gene ontology (GO) analysis of identified DEGs was performed to reveal enriched pathways.

#### Pipeline 1

Fastq files for each sample were aligned to the GRCh38 genome (Gencode, release 37) using HISAT2 (v2.0.1b)^15^ with default parameters. Quality control for each sample was performed using FastQC (version 0.11.9)^16^ and RSeQC (version 4.0.0)^17^. Counting reads was done using featureCounts^18^. Differential expression analysis was done using DESeq function from DESeq2 with default settings^19^ in R (version 4.0.3). The following design was used in the analysis: gene ~ age + group + RIN + time of tissue collection (ttr). Detected differential expression of genes was considered significant at p.value < 0.05 and fold change (FC) threshold > 1.5.

#### Pipeline 2

Adapters were removed by Cutadapt (version 3.4). Fastq files for each sample were aligned to the GRCh38 genome (Gencode, release 37) using HISAT2 (version 2.2.1)^15^ with default parameters to build an index of the reference genome and mapping reads to the genome. Quality control for each sample was performed by FastQC (version 0.11.9)^16^ and RSeQC (version 4.0.0)^17^. Counts of the number of sequencing reads mapping to each gene after the alignment step were conducted using the htseq-count function from the HTSeq framework (version 0.6.1)^20^. Gene differential expression analysis of two groups (two biological replicates per condition) was performed using the DESeq2 package (version 1.30.1)^19^ in R (version 4.0.3). The following design was used in the analysis: gene ~ age + group + RIN + ttr. Detected differential expression of genes was considered statistically significant at p.value < 0.05 and FC threshold > 1.5.

#### Pipeline 3

Ambiguous and low-quality bases were removed from the files obtained during the FastQ sequencing. The bases were removed with AdapterRemoval V2^21^. The trimmed files were aligned to the human reference genome GRCh38 and GRCh38.92 gene annotation using RSEM with the “rsem-prepare-reference” and “rsem-calculate-expression commands”^22^, and the “-star” option was also used to generate STAR indices^23^. The resulting pseudocounts were normalized using the TMM algorithm implemented in the R “edgeR” package^24^, as well as the “calcNormFactors” command and the CPM algorithm implemented in the R “limma” package, with the “voom” command^25^. To identify differential expressions, the normalized reads were processed using the commands “voom” (estimating the ratio of mean to variance, determining weights for observations), “lmFit” (creating a linear model describing observations), and “eBayes” (determining model parameters) from the R “limma” package^25^. In the linear model, the outcomes of the disease, age, time from the moment of tissue collection to isolation, and RNA integrity index were considered. DEGs were selected according to the following criteria: FC threshold > 1.5 and p-value moderated t test limma < 0.05.

#### Gene ontology enrichment analysis

Gene ontology (GO) enrichment analysis with biological processes terms was performed using Clue GO version 2.5.5^26^ and Cluepedia version 1.5.5^27^ for Cytoscape version 3.6.1 and was conducted for DEGs obtained using three pipelines. Significantly enriched terms were selected using the one-sided hypergeometric test with FDR correction (*p* < 0.01). Term groups were selected by ClueGO based on the number of common genes/term (> 40%). For enrichment, only terms of at least level 3 and no more than level 8 were considered, with which at least 3 DEGs were associated and with which the total number of DEGs associated was at least 4% of all genes associated with the term. All genes for which at least three reads were identified were used as a background for enrichment analysis.

## Results

### Differential gene expression analysis using DESeq2 and Limma/voom

We obtained PBMCs from each patient with severe COVID-19: five patients who survived (survivors) and five patients who died from infection in the ICU (nonsurvivors). Variation across biological replicates was low with Spearman correlation values within replicates for the two groups ranging from 0.86 to 0.89. When applying a threshold of FC > 1.5 and p.value.adjusted < 0.05. With FDR multiple testing corrections, no DEGs were identified. After applying a threshold of FC > 1.5 and p.value < 0.05, we identified a total of 429 DEGs (292 upregulated and 156 downregulated genes) using Pipeline 1, 345 DEGs (266 upregulated and 79 downregulated genes) using Pipeline 2, and 306 DEGs (191 upregulated and 115 downregulated genes) using Pipeline 3 in survivors compared with nonsurvivors among patients with severe Delta SARS-CoV-2 COVID-19 enrolled in this study. Lists of all DEGs of the three pipelines are available in Supplementary Table 1. Seventy-seven (60 upregulated and 17 downregulated) common DEGs were identified among all three pipelines (Supplementary Table 2). DEGs were considered to be common between pipelines if and only they were identified to be significantly differentially expressed in each pipeline.

### Enrichment gene ontology analysis

**Fig. 2:**
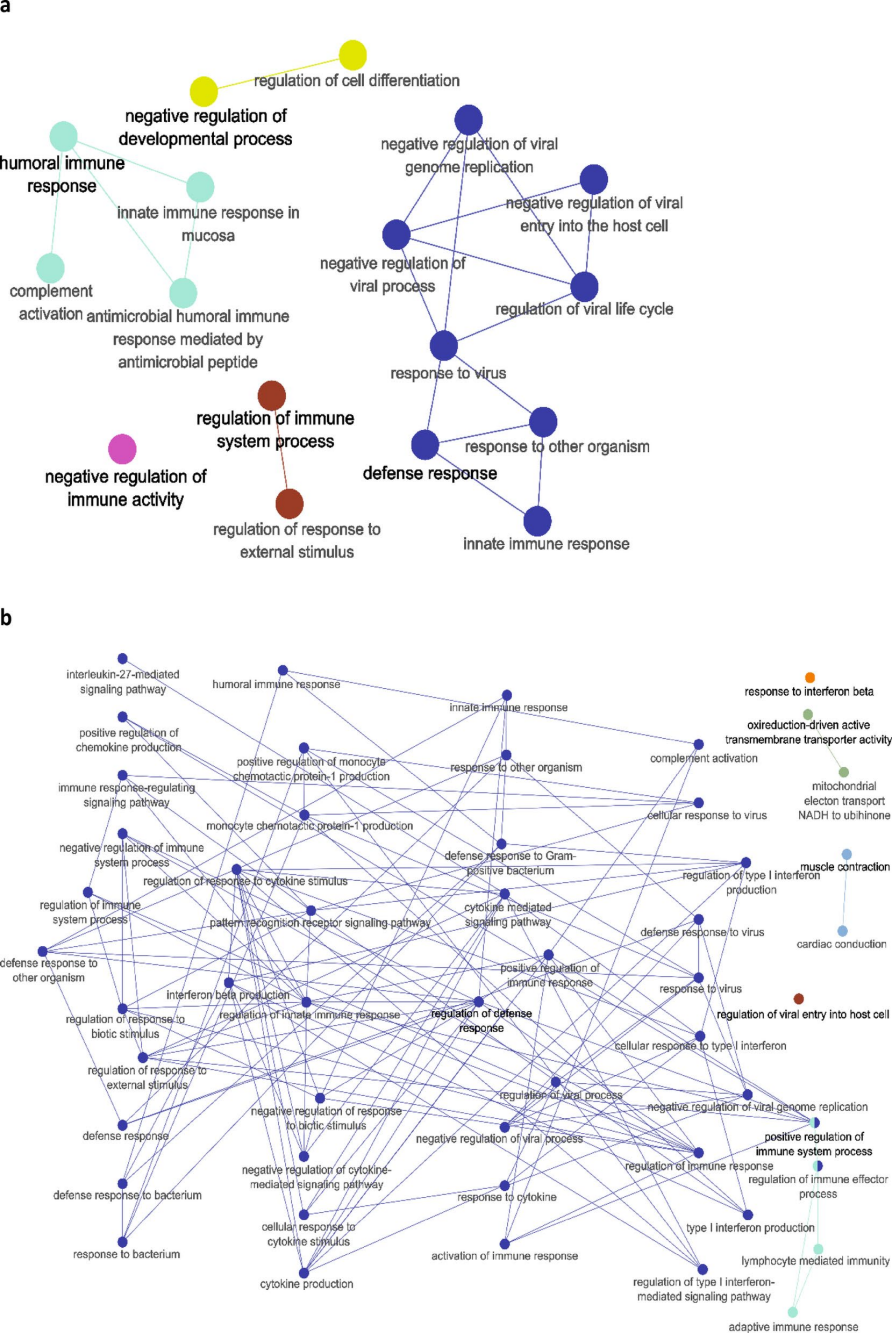

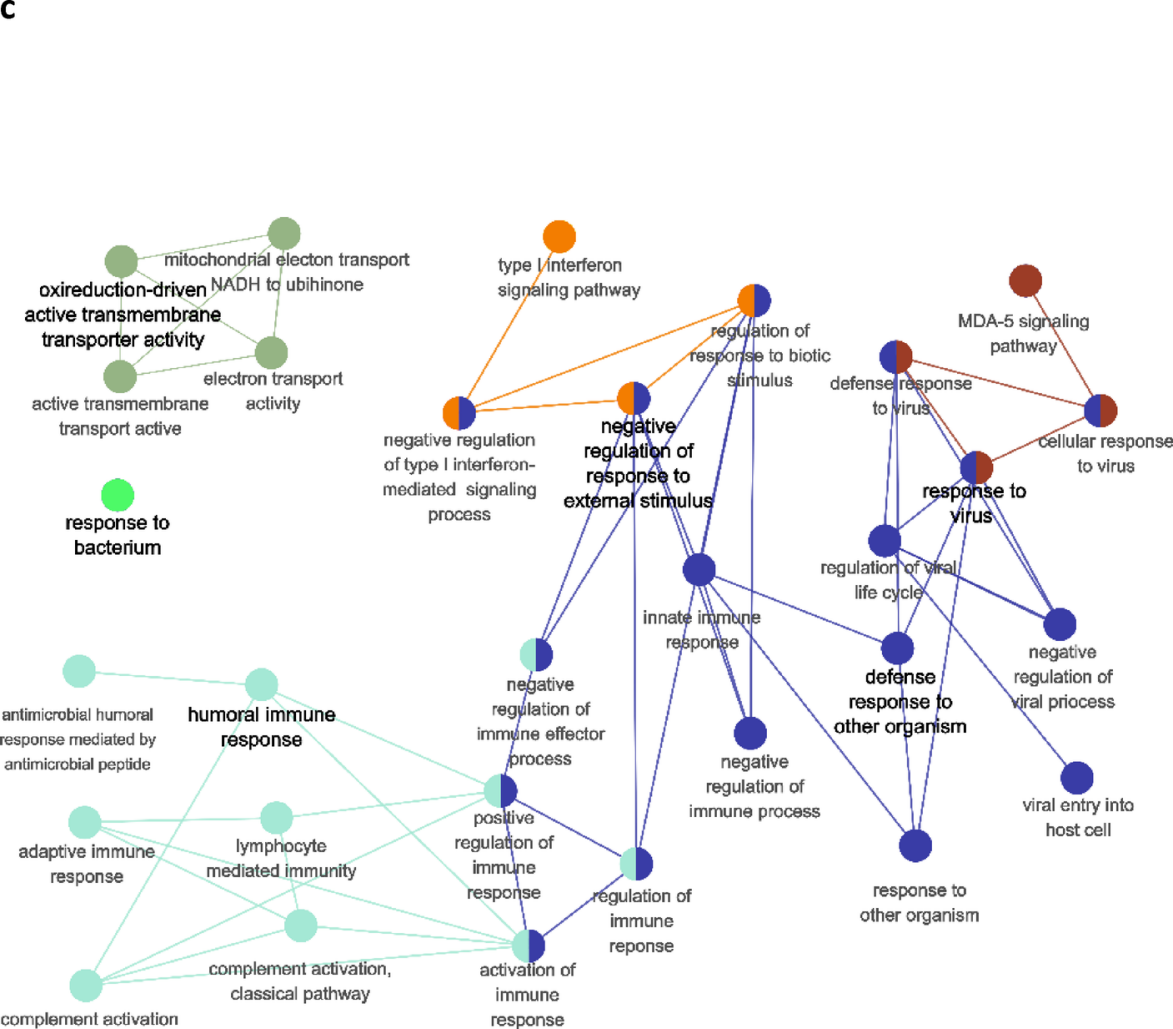
GO term-term network analysis of the significantly enriched GO terms in differently expressed genes in survivors compared to nonsurvivors (multiple test correction by BH). (**A**) Pipeline 1; (**B**) Pipeline 2; (**C**) Pipeline 3.

To identify biological pathways in Delta SARS-COV-2 COVID-19 patients systematically, we performed GO enrichment analysis of significantly up- and downregulated genes for DEGs identified in each pipeline, as well as separately for the genes, common between all three pipelines. The functional modules were identified as mutually overlapping gene sets clustered together and named using GO hierarchical structure terms. A GO term enrichment was conducted for these genes. We considered “metabolic process” terms with *p* < 0.05 (Bonferroni corrected hypergeometric test) and all types of GO-term-to-gene connections. Significantly enriched terms are presented in Fig. 2 and Supplementary Table 3. Most significantly enriched terms were related to the processes of inflammation, innate immunity and viral replication. In Pipeline 1, such terms as GO:0070106 (interleukin-27-mediated signaling pathway), GO:0071639 (positive regulation of monocyte chemotactic protein-1 production), GO:0071605 (monocyte chemotactic protein-1 production, GO:0045071 (negative regulation of viral genome replication), and GO:0035456 (response to interferon-beta). In the case of Pipeline 2, terms with the lowest p.val were related to natural immunity and inflammation, such as GO:0002227 (innate immune response in mucosa), GO:0046597 (negative regulation of viral entry into host cell), GO:0006956 (complement activation), GO:0061844 (antimicrobial humoral immune response mediated by antimicrobial peptide), and GO:0045071 (negative regulation of viral genome replication). However, some unexpected terms were significantly enriched in Pipeline 2—muscle contraction (GO:000693) and oxidoreduction-driven active transmembrane transporter activity (GO:0015453). The latter term was also significantly enriched in Pipeline 3.

In the case of Pipeline 3, most clusters also were related to the immune response—GO:0006958 (complement activation, classical pathway), GO:0039530 (MDA-5 signaling pathway), GO:0006956 (complement activation), GO:0006120 (mitochondrial electron transport, NADH to ubiquinone), and GO:0060339 (negative regulation of type I interferon-mediated signaling pathway).

**Fig. 3:**
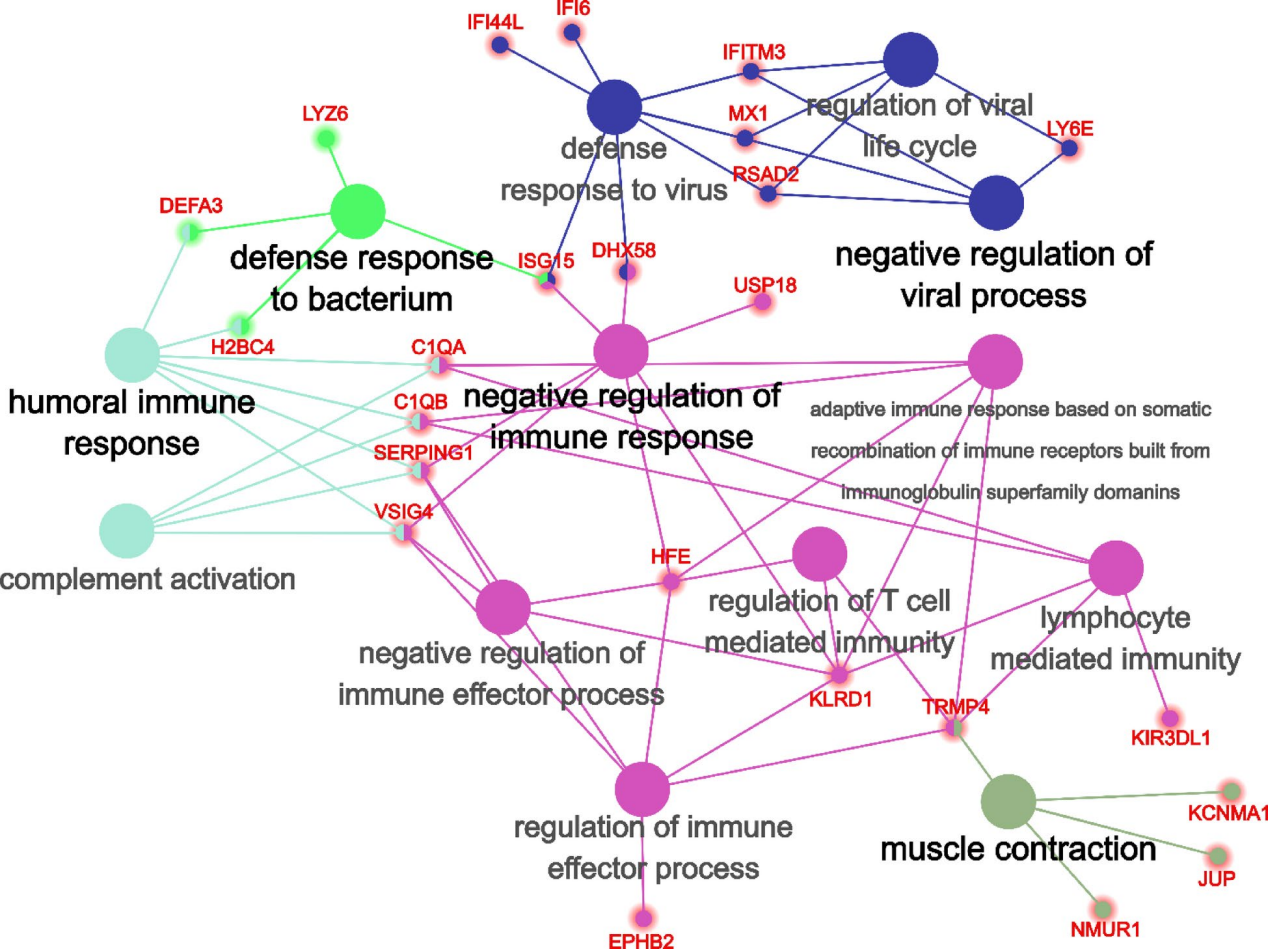
GO gene–term network analysis of the top enriched GO terms in differentially expressed genes in survivors compared to nonsurvivors (multiple test correction by BH) in overlapping DEGs between all pipelines. Halos around genes indicate the direction of differential expression, with a red halo denoting increased expression in the survivor group as compared to the nonsurvivor group and a green halo denoting reduced expression in the survivor group as compared to the nonsurvivor group.

Overlapping DEGs (77 total, 60 upregulated, 17 downregulated) in PBMCs of survivors compared with nonsurvivors between the three pipelines were identified and used for further analysis. The overlapping genes are presented in Supplementary Table 2. All but two (75 out of 77) the overlapping DEGs between the three pipelines had the same direction of fold change. Next, we conducted GO enrichment for 77 DEGs, common between three pipelines (Fig. 3). Fourteen significantly enriched terms were identified, 13 of which are related to aspects of immune response and viral processes (Fig. 3, Supplementary Table 4). A term–gene network has been constructed based on the relation of terms and genes from GO. Five groups were identified based on common DEGs among the terms.

Group one consists of six terms—GO:0050777 (negative regulation of immune response, the “main term” with the highest significance/lowest p value), GO:0002709 (regulation of T cell-mediated immunity), GO:0002697 (regulation of immune effector process), GO:0002698 (negative regulation of immune effector process), GO:0002449 (lymphocyte-mediated immunity), and GO:0002460 (adaptive immune response based on somatic recombination of immune receptors built from immunoglobulin superfamily domains). Based on the main term and intersection of the terms, this group can be related to negative regulation of immune response. It is worth noting that every gene in this group is upregulated in survivors compared to nonsurvivors.

Group two consists of three terms—GO:0048525 (negative regulation of viral process), GO:0051607 (defense response to virus), and regulation of GO:1,903,900 (regulation of viral life cycle). The terms of this group are related to negative regulation of viral processes.

Group three consists of two terms—GO:0006956 (complement activation) and GO:0006959 (humoral immune response). It is worth noting that the term “complement activation” is the most significantly enriched of all the terms and has the highest percentage of related genes differentially expressed.

Both groups four and five consist of a single term—GO:0006936 (muscle contraction) and GO:0042742 (defense response to bacterium), respectively.

Based on this enrichment, gene *ISG15* is especially interesting, since it is related to three different GO term groups at the same time. Its expression in increased 2.8-fold in survivors compared to nonsurvivors. It is also notable as one of four genes related to five terms at the same time—GO:0050777 (negative regulation of immune response), GO:0048525 (negative regulation of viral process), GO:1,903,900 (regulation of viral life cycle), GO:0051607 (defense response to virus), and GO:0042742 (defense response to bacterium).

**Fig. 4:**
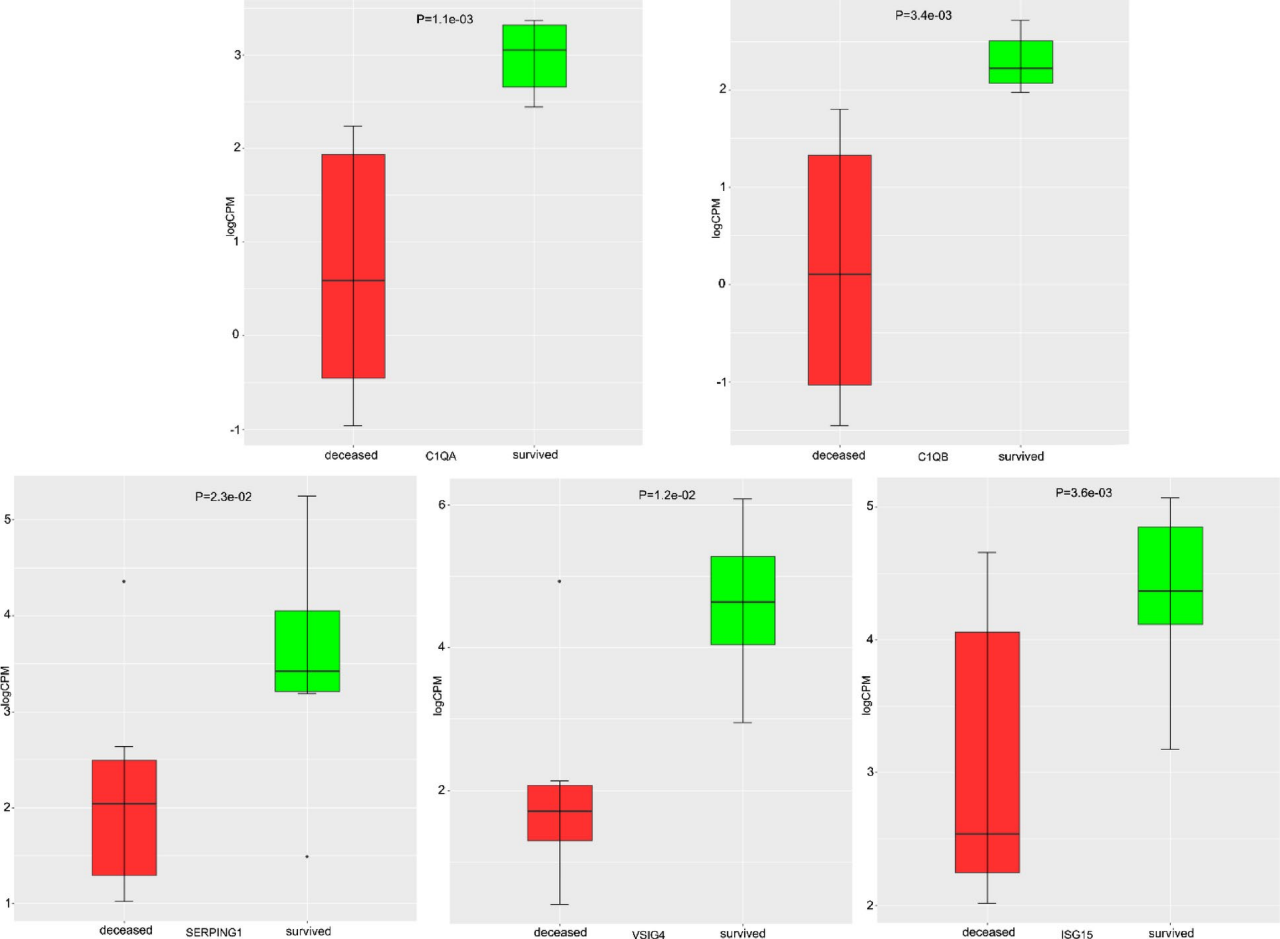
Average (between three pipelines) expression in logarithm of counts per million (logCPM) per group. Red color denotes gene expression in the nonsurvivor group; green color denotes gene expression in the survivor group.

Eight genes are related to four and more significantly enriched terms—C*1QB*, *C1QA*, *ISG15*, *SERPING1*, *VSIG4*, *KLRD1*, *TRPM4*, and *HFE.* Expression of all these genes is increased in survivors compared to nonsurvivors. Genes of the first component of the complement system—C*1QB* and C1QA—were among the most overexpressed (a more than 4.5-fold increase). The same is the case for the gene encoding the immunoregulatory protein VSIG4. Average expression levels (in CPM) of five of these genes per group can be seen in Fig. 4.

## Discussion

Our study has identified a set of genes, the differential expression of which is associated with survival outcomes in severe COVID-19, particularly through their involvement in negative regulation of immune response, complement activation, and the defense response to the virus. To achieve this, we have conducted transcriptomic analysis of PBMCs derived from patients with severe SARS-CoV-2 Delta strain induced pneumonia and identified a number of genes differentially expressed between survivors and nonsurvivors in the period of 30 days after hospitalization in the ICU. To elucidate the role of identified DEGs in determination of survival outcome, we have conducted Gene Ontology Biological Processes (GO: BP) term enrichment. Most of the terms significantly enriched by DEGs were related to various immune and antiviral processes. A term–gene network has been constructed, based on the relation of terms and genes from GO. Significantly enriched terms can be split into five groups based on common genes—negative regulation of immune response (group 1), negative regulation of viral processes (group 2), humoral and complement-system activation (group 3), defense response to bacterium (group 4), and muscle contraction (group 5). Next, we will analyze potential role of significantly enriched gene ontology biological processes in COVID-19 and identify some key genes based on their position in the term–gene network and the current understanding of their role in the pathophysiology of COVID-19 and other diseases.

However, before this, it is worth mentioning that the small sample size impacts the generalizability of the study’s findings, especially in what concerns differential expression of particular genes. Since Gene Ontology enrichments impose additional levels of statistical testing on the data, we believe that identification of biological processes as a whole in the outcome of COVID-19, and less so individual DEGs, is the main takeaway of the study. Differential expression of identified key genes needs to be further validated on larger independent cohorts in order to confirm their role as important determinant in COVID-19 outcome and potential therapeutic targets.

### The dual role of immune regulation in COVID-19

It has been previously established that the role of immune response in COVID-19 is twofold—on one hand, strong and timely activation of the immune system is necessary to prevent viral replication and stop the infection, but, on the other hand, the immune system hyperactivation that develops in the severe cases of COVID-19 contributes to its severity and lethality^28^. Data obtained in our own study further contribute to this twofold picture. The significant enrichment of terms related to negative regulation of immune response and the fact that most of these genes are upregulated in survivors imply that the positive outcome of severe COVID-19 is partially based on downregulation of the immune system. However, significant enrichment of terms related to negative regulation of viral processes shows that strong antiviral immune response is still necessary for survival in severe COVID-19 cases. SARS-CoV-2’s ability to evade the immune system and its ability to cause reduced interferon response are well documented^29^. The differential expression of genes related to these terms in the survivor group underlines the crucial role of effective immune response in counteracting COVID-19. The importance of interferon response is further stressed by group 2 terms being specifically related to three interferon induced DEGs—I*FI6*, *IFITM3*, and *IFI44L—a*ll of which are overexpressed in survivor group. It is further worth noting that only two DEGs are related to both groups 1 (negative regulation of immune response) and 2 (negative regulation of viral processes), with most being exclusively related to either group, suggesting that different sets of genes are involved in negative and positive components of immune system regulation. Taken together, these data points illustrate that the balance between immune activation and regulation appears critical for survival in severe COVID-19. While robust antiviral defense mechanisms are necessary, excessive immune responses, such as the dysregulated cytokine production observed in patients with severe and critically severe cases of COVID-19^30^, can exacerbate the disease and cause additional harm.

Putting the terms “humoral response” (as a part of adaptive immunity) and “complement activation” (as a part of natural immunity) into a single group is most likely based on the involvement of the common genes, associated with both groups 1 and 3—S*ERPING1*, *VSIG4*, *C1QB*, and *C1QA*. Before discussing these genes, however, it is worth examining the genes that are related to humoral response but not to complement activation. Both of them, *DEFA3* and *H2BC4*, are also simultaneously related to the term “defense response to bacterium,” and these genes are most likely the reason behind enrichment of the “humoral response” term. Enrichment of the term “defense response to bacterium” appears at first glance to be a result of an error. However, upon further examination, this enrichment could have important implications for its role in the outcome of COVID-19. Genes *LYZ*^31^, *DEFA3*^32^, and *H2BC4*^33^ encode proteins with well-documented antimicrobial properties. Among them, *DEFA3* and *H2BC4* are related to both terms humoral immune response and defense response to bacterium, while *LYZ* is related exclusively to defense response to bacterium. All three genes of these are upregulated in the group of nonsurvivors compared to survivors. Most of the genes used in GO enrichment are the opposite—upregulated in survivors compared to nonsurvivors (60 out of 77)---which makes it highly unlikely that this happened by chance. This may imply that downregulation/absence of upregulation of the antimicrobial and humoral response may play an important role in a positive outcome of COVID-19.

### The role of ISG15 as a potential key gene in determining COVID-19 outcomes

In the gene–term network (Fig. 3), gene *ISG15* occupies a key position, as it is the only gene that connects three different groups (1, 2, and 4) and is related to five different significantly enriched GO terms. *ISG15 *encodes interferon-inducible ubiquitin-like protein^34^, which binds covalently to various substrates originating from both host cell and virus in a process called “ISGylation.” ISGylation plays an important role in inhibition of viral replication. The precise role and fate of ISGylated proteins are unknown; however, several possible mechanisms have been found. ISGylation can affect host protein localization, as in the case of filamin B^35^, and it also could disrupt aggregation of viral protein complexes, as was observed in interaction of ISG15 with influenza B virus and human papilloma virus^36^. ISG15 can also disrupt the egress of virus from host cells, as has been shown using the example of interaction of ISG15 with human immunodeficiency virus 1 (HIV-1), in which release of HIV-1 virions, but not production of HIV-1 proteins, were impeded by overexpression of ISG15^37^. Besides interacting with various proteins through ISGylation, unconjugated ISG15 can act as a cytokine. Extracellular ISG15 acts as a ligand for integrin ITGAL^38^, interacting with it and stimulating release of IFNγ and IL10 from NK cells through that interaction, with expression of IL10 being especially notable as an important factor in COVID-19 prognosis^30^.

Previously, activation of *ISG15 *expression was identified in expressional profiling of nasopharyngeal swabs of patients infected with COVID-19^39–41^. On the other hand, in vitro SARS-CoV-2 infection did not cause an increase in *ISG15 *mRNA production, which could be explained by specific properties of the used cell culture^42^. The important role of *ISG15 *can be also emphasized by the concentration and activity of ISG15 in a cell regulated by SARS-CoV-2. Papain-like proteinase PLpro plays an important role in this regulation. In SARS-CoV-2, PLpro is both necessary for normal processing of viral proteins and in regulation of posttranslational modification of host proteins, primarily ISG15, whereas in other coronaviruses it is primarily employed to prevent ubiquitination of cellular and viral proteins^43–45^. The main targets of PLpro are complexes of ISG15 with IRF3 (interferon responsive factor 3, regulator of type 1 interferon response) and MDA5 (a protein from the family of RIG-I-like receptors, stimulating the synthesis of interferon and proinflammatory cytokines when binding to viral RNA)^46,47^. This adaptation, allowing SARS-CoV-2 to target specifically ISG15 and ISGylated proteins, demonstrates the vital importance of ISG15 in antiviral immunity and suggests that increased expression and, consequently, activity of ISG15 play a crucial role in determining the outcomes of SARS-CoV-2 infection. Increased levels of *ISG15* mRNA in the survivor group may lead to increased presence of ISG15 protein and, in turn, allow some of it to avoid inactivation by SARS-CoV-2. Based on our data and previous research, we consider overexpression of *ISG15* to be a key event in determining a positive outcome of severe COVID-19 and believe it to be a valuable target for therapeutic intervention.

### The complement system in COVID-19

Based on the raw number of related terms in the enrichment, eight key genes can be identified—C*1QB*, *C1QA*, *ISG15*, *SERPING1*, *VSIG4*, *KLRD1*, *TRPM4*, and *HFE*. Four of these genes are related to both groups 2 and 3 and to the terms “complement activation” and “negative regulation of immune response,” as well as the aforementioned “humoral response”---*C1QA*, *C1QB*, *SERPING1*, and *VSIG4*. Going further, we would like to focus our attention on these four genes and on their role and the wider role of the complement system in COVID-19. Also, it is worth noting that all four of these genes are upregulated in survivors compared to nonsurvivors.

The complement system plays a role in antiviral infections by opsonizing viruses and virus-infected cells, directly neutralizing viruses outside of cells, lysing virus-infected cells, and inducing virus-specific immune and inflammatory responses^48^. The complement system can be activated through three possible pathways—classical, nonclassical, and lectin. Generally, it is considered that all the pathways of complement system activation lead to the same protein cascade and activation of the same effector mechanisms. However, based on our own and previously obtained data, it appears that the pathway of complement system activation could be important for determining outcomes of COVID-19. As previously mentioned, significantly increased expression of genes *C1QA* and *C1QB*, encoding both subunits of the first complement system component (related specifically to the classical pathway of complement activation), was observed in our comparison. Currently, it is not completely clear how interaction with SARS-CoV-2 activates the complement system. It has been shown previously that all of the major SARS-CoV-2 proteins (S, N, M, E) interact with the first component of the complement system, engaging the classic pathway of complement system activation^49^. However, additional data exist that contradict these finding and implies that SARS-CoV-2 proteins do not directly bind to C1Q, instead activating the complement through the lectin pathway^50^. Complement system proteins deposits are found in various tissues of COVID-19 patients, e.g., alveolar, kidney, and liver capillaries. However, most of those deposits consist of complement system proteins related to a nonclassical complement-system activation pathway^51^. A retrospective examination of concentration of several complement system components (C1Q, C3, C4, and C5) in a cohort of 74 patients with COVID-19 has demonstrated that a high level of expression of nonclassical and lectin complement system activation pathway proteins are linked to negative outcomes of COVID-19, while no analogous connection was found in the case of classical pathway^52^. It has also been shown that in a cohort of 71 patients, levels of C1q in serum were decreased in severe cases of COVID-19 when compared with patients with mild COVID-19^53^. While not all changes in expression that can be observed in comparisons of mild and severe cases must necessarily also be present in comparisons of survival and nonsurvival outcomes, the assumption can be made that transcriptomic profiles of patients with mild cases of COVID-19 will be closer to those of surviving patients and, correspondingly, the transcriptomic profiles of patients with severe cases will be closer those of patients with terminal outcomes. Under this assumption, we can observe that our results are in accordance with previously obtained data, since we have observed an increase in the expression of both subunits of first complement component in the survivor group. Taken together with previously published data, increased expression of *C1QA* and *C1QB *in the survivor group implies that activation of the complement system via the classical pathway may contribute to a positive outcome of the disease. However, whether this relationship is casual in nature or is an association should be interpreted carefully, since it has also been shown that the concentration of С1q, unlike that of IL6 and IL10, is not an independent predictor of clinical outcomes, being instead dependent on the concentrations of other components of the “cytokine storm”^30^.

### The twofold role of the complement system in determining COVID-19 outcomes

Previously, the twofold role that immune system regulation plays in COVID-19 pathogenesis has been mentioned. The role of the complement system in SARS-CoV-2 pathogenesis also fits a twofold paradigm. On one hand, hyperactivation of the complement system has long been implicated in development of respiratory failure in both COVID-19^54^ and other diseases^55^, and targeting the complement system as an intervention appears a possible strategy for improving outcomes in severe COVID-19^56,57^. On the other hand, the complement system plays an indispensable role in combating infectious diseases^58^, and inhibiting it as an intervention opens the door to a significant increase in the risk of treatment-induced infection-related side effects^56,57^ due to disruption of its role in immune response. Several putative reasons for the twofold role of the complement system in severe SARS-CoV-2 pathogenesis can be posited. The first is timing, with early activation being beneficial and the later role of the complement becoming detrimental^48^; the second being the “Goldilocks zone”^58^, with both too much activation and too little being detrimental; and the third one being that the particular pathway of activation determines whether complement activation will be detrimental or beneficial. Our own data could be considered as supporting the third hypothesis, seeing as we have identified that an increase in the expression of components of the complement system specifically involved in only the classic complement activation pathway is connected to survival outcomes. Previous research has also shown that outcomes of COVID-19 are associated with changes in concentration of components related to particular pathways of complement-system activation, but not just increase or decrease in activation of system overall^51–53^. Based on our data and previous research, increased expression of *C1QA* and *C1QB* could to be involved in pathogenesis and lethality of COVID-19, and could be considered a molecular marker of milder cases, and stimulation of their expression could be a possible option for therapeutic intervention. However, role of the complement system in SARS-CoV-2 is complex, and therapeutic interventions involving complement system are not without risk. Further research is required in order to better understand how complement system affects outcomes in SARS-CoV-2 infections.

### VSIG4 and SERPING1—Potential key regulators of the complement system in COVID-19

Another key upregulated gene related to negative regulation of the immune system, humoral system, and complement activation is *VSIG4*, a gene encoding V-set and Ig domain-containing four proteins, also known as the “complement receptor of the immunoglobulin superfamily” (CRIg). This gene is expressed by macrophages and dendritic cells, participating in ingestion of complement-opsonized particles. VSIG4 also inhibits activation of a nonclassical complement pathway by binding to C3b^59,60^, leading additional credence to the idea that the particular pathway of complement activation is one of the factors determining survival outcomes in COVID-19 patients, since its overexpression is linked to survival in our data. Besides participating in phagocytosis of complement-opsonized particles and complement system regulation, engagement of *VSIG4*with its ligands in macrophages also causes immunosuppressive and anti-inflammatory effects^61^.

The role of *VSIG4*in COVID-19 pathogenesis is not fully understood. However, data points exist that point to its increased expression being both possibly detrimental and possibly beneficial. Reduced expression of VSIG4 leading to inhibition of anti-inflammatory activity was previously described in СOPD (chronic obstructive pulmonary disease) as an important factor in its pathogenesis^62^. Since, based on our GO: BP enrichment, we consider negative regulation of the immune system being important in survival for severe COVID-19, its previously demonstrated immunosuppressive and anti-inflammatory activity could be beneficial. On the other hand, overexpression of this protein is considered an unfavorable factor in ARDS (acute respiratory distress syndrome)^63^ and lung cancer^64,65^, which could also mean its overexpression could be a detrimental factor in COVID-19, seeing how respiratory failure is one of the more dangerous symptoms in severe cases of COVID-19. Therefore, increased *VSIG4* can be both a detrimental and beneficial factor in severe COVID-19. Its overexpression in our data suggests that it could play a protective role in severe COVID-19 through modulation of the complement system response, inhibition of alternative pathway of complement activation and anti-inflammatory and immunosuppressive activity as a receptor. Based on its position in gene–term network and its role in several important processes in COVID-19 pathogenesis, we consider *VSIG4* to be one of the key genes and a potential biomarker and target for therapeutic intervention.

The final complement-system related differentially expressed key gene is *SERPING1. SERPING1*encodes C1-INH, a serine protease inhibitor (serpin), which plays a role in inhibition of the innate immune responses and complement system activation^66^. C1-INH is capable of inhibiting all three pathways of complement activation and has shown the capability of limiting the complement and inflammation-related damage in multiple injury models^67,68^. C1-INH inhibits those pathways via forming covalent bonds with C1r and C1 s (classic pathway), MASP-1 and MASP-1 (lectin pathway), and C3b (alternative pathway)^66^, and it is worth noting that, since this is a posttranslational mechanism, an observed increase of *C1QA* and *C1QB* expression on the transcriptional level may be not connected with increased activity of C1q. Being an interferon-induced gene, *SERPING1*could be posited to play a role of negative feedback mechanism, limiting immune system hyperactivation and, therefore, improving outcomes^66^, which is especially important, considering the propensity of severe COVID-19 to cause immune-related damage to various tissues. *SERPING1*is implied to play a role in COVID-19 pathogenesis with its expression being shown to be increased compared to healthy individuals in reanalysis of three cases of from 2020^69^. It has also been shown that the T allele of variant rs78958998 is associated with increased risk of viremia in COVID-19 cases^70^. Overall, its beneficial role as a negative feedback mechanism against runaway immune processes fits very well with current understanding of the possible role of immune-mediated damage in severe COVID-19.

Currently, to our knowledge, no therapies for COVID-19 or other infectious diseases have been developed that specifically target *ISG15*, *SERPING1*, or *VSIG4 *genes. As an IFN-induced gene, ISG15 can be upregulated by introduction of a wide variety of compounds, such as type I IFN, double-stranded RNA, lipopolysaccharide, or all-trans-retinoic acid^71^. However, the effect of such compounds is not specific to inducing *ISG15 *expression, and they have a wide variety of effects on many different biological processes and induce a variety of different responses. IFN treatment appears to be the most promising among those, however evidence for efficacy and safety of IFN treatments in SARS-CoV-2 is mixed^72^, leading to outcomes that are better than control in some and worse than control in other reports. Currently, treatment of hereditary angioedema with recombinant SERPING1 (Ruconest, Salix Pharmaceuticals) is approved in some jurisdictions^73^, and it could be interesting to investigate it for efficacy in SARS-CoV-2 cases, although it is important to be mindful of how angioedema pathophysiology is different from that of COVID-19. As for *VSIG4*, anti-VSIG4 antibodies have been demonstrated to be able to induce immune response in vitro, in vivo, and ex vivo assays, but the relevance of these findings for SARS-CoV-2 remains to be shown, especially considering the fact that, in the original paper presenting those findings, the goal was to suppress activity of VSIG4, not activate it^74^.

### Determinants of outcome in the Delta vs. Alpha variant of COVID-19

Next, we would like to compare our data obtained in this study to the results of our own previous investigations of COVID-19. Previously, in a different paper, we have demonstrated that a lethal outcome in COVID-19 caused by SARS-CoV-2 Delta variant infection was associated with increased levels of pro-inflammatory cytokines^75^. However, in another paper, we have shown a downregulation of LDL particle-receptor pathway activity in ICU patients who survived severe COVID-19 infection^14^, which was in agreement with results obtained in independent studies, including a meta-analysis of 22 published works^76–78^. It is worth considering why those findings have not been replicated in the present study. The methodology of the present study and the 2021 study are very similar, from patient selection to the analysis of differential expression. One of the significant differences is in the variant of SARS-CoV-2 causing the disease. The Alpha variant was the most widespread variant in St. Petersburg during the patient cohort collection of the 2021 study^14^. The Alpha variant is characterized by seven missense mutations (N501Y, A570D, D614G, P681H, T716I, S982 A, and D1118H) and three deletions (69/70 del and 144 del) in spike protein S. The Delta variant is characterized by a different set of mutations (T19R, G142D, Δ157–158, L452R, T478 K, D614G, P681R, and D950 N), and the identified differences could affect the interaction of S protein with ACE2 and immune-system proteins^10,79,80^. This could lead to both differences in virus infectivity and activation of different metabolic pathways in pathogenesis of COVID-19. The role of particular missense variants in interaction with cellular proteins is not clearly understood. For example, there have been no mutations identified in the gene encoding PLpro between Delta and Alpha variants of SARS-CoV-2^81,82^. However, no differences in the expression of *ISG15 *between survivor and nonsurvivor groups have been identified in our data in the case of severe COVID-19 caused by infection by the Alpha variant of SARS-CoV-2. This suggests that the nature of our findings may be variant-specific. However, despite our best effort, several confounding variables are present, and could not be fully accounted for, as will be discussed further in the Study Limitations section. Therefore, interpretation of strain-specificity in our findings needs to be done cautiously. Nevertheless, the differences between the results of this and the previous study are evident. Significantly enriched biological processes in this study are almost exclusively related to the immune system, whereas there was a high number of metabolism-related significantly enriched processes in our previous study^14^. One possible explanation of such differences would be the role of evolving immune evasion of SARS-CoV-2. With SARS-CoV-2 constantly getting better at avoiding the immune system^83^, the role of subtle differences of immune system regulation and activity in determining outcomes of COVID-19 should also increase.

Further study of patients infected by different variants of SARS-CoV-2 may elucidate the role of the variant- specific aspects of pathogenesis of COVID-19. We believe that continuous research of COVID-19 cases caused by different variants of constantly evolving SARS-CoV-2 is important. To accomplish this, our suggestion would be to attempt to conduct periodic studies of SARS-CoV-2 infection using various methods, such as RNA-seq, while keeping the design of each study as constant as possible. This would allow keeping as many of the confounding variables the same as possible and to investigate the variant-specific aspects of pathogenesis of COVID-19. In our work, taking together the present paper and a previous publication of a similarly designed investigation of COVID-19 infection caused by the Alpha variant^14^, we have attempted to do exactly that. Fortunately, thanks to the spread of vaccinations, improving standard of care, and reduced load on health-care systems, number of lethal cases of COVID-19 has significantly gone down. However, this impairs our ability to adhere to the same study design in the future (comparison of survivors and nonsurvivors), which suggests that, going forward, in a series of experiments on different strains and variants of SARS-CoV-2, we will need to employ different comparisons, such as, e.g., severe versus mild cases.

## Conclusion

The transcriptomic analysis of PBMCs in patients with severe COVID-19 identified a number of genes differentially expressed between survivors versus nonsurvivors. Enrichment has been identified of several groups of significantly enriched GO biological-process terms related to various immune processes. Based on the significantly enriched terms and their related genes, several processes that may be important for surviving severe COVID-19 have been identified, such as upregulation of genes of the classical complement activation pathway, upregulation of genes related to negative regulation of the immune process, and upregulation of genes related to negative regulation of viral processes and downregulation of genes related to defense against microbial infection.

Several key genes have been identified based on this enrichment—C*1QB*, *C1QA*, *ISG15*, *SERPING1*, *VSIG4*, *KLRD1*, *TRPM4*, and *HFE*. Gene *ISG15*, increased expression of which can play a role in response to viral protease PLpro, is especially notable. The identification of key immune-modulating genes like *ISG15*, *SERPING1*,* C1QA*,* C1QB*, and *VSIG4* opens avenues for therapeutic approaches that aim to modulate the immune response and improve outcomes in severe COVID-19 cases via upregulation of expression of these genes. However, due to the small sample size used in the study and the use of p.values, not corrected for multiple testing, we believe that findings regarding particular differentially expressed genes should be considered to be preliminary and require confirmation on larger independent cohorts.

Previously, we have shown that downregulation of the LDL particle-receptor pathway activity plays a role in ICU patients that survive severe COVID-19 infection caused by the Alpha variant of SARS-CoV-2. On the other hand, most DEGs in comparison of survivors versus nonsurvivors of severe COVID-19 caused by the Delta variant are related to immune response. Such differences may be related to mutations in the structure of a viral protein, which lead to changes in pathogenesis of severe COVID-19.

### Study limitations

1. The Delta variant of SARS-CoV-2 appears to be either extinct or almost extinct, with new studies of the Omicron variant and its subvariants being necessary to elucidate the differences between survivors and nonsurvivors in pathogenesis of severe COVID-19.
2. Despite our best efforts at replicating the design of the previous study, several changes have been made, such as in the number of included patients being changed from five versus three in a 2021 study to four versus five in the current study, and the number of technical replicates in 2021 of two being changed to one in the current study, which may impact the comparability of the two works.
3. Apart from changes in design, numerous changes to factors outside of our control have occurred since the previous study, with probably the most important being the ubiquity of vaccinated patients among ICU patients and changes in the ICU treatment protocols that are used. All of this makes identifying the true reasons behind the differences between the two studies almost impossible.
4. It is worth noting that the small sample size (five versus five) and use of p.values without multiple test corrections impacts the interpretability of our study. Due to the fact that enrichments pass through additional level of statistical tests, significantly enriched biological processes are probably the most generally applicable part of our results, whereas significant changes in the expression of particular genes (even the ones found in all three pipelines) need to be confirmed on independent cohorts before considering them for further examination or use.

## Electronic supplementary material

Below is the link to the electronic supplementary material.

Supplementary Material 1

Supplementary Material 2

Supplementary Material 3

Supplementary Material 4

Supplementary Material 5

## Data Availability

Raw FASTQ files and processed read counts are available from the Gene Expression Omnibus (GEO), accession no. GSE272392. In order to obtain additional data for this study, please contact the corresponding author at email provided on the title sheet.
