# Correction: Transcriptomic analysis of differential expression between surviving and nonsurviving patients infected by the SARS-CoV-2 Delta variant

**DOI:** 10.1038/s41598-025-23927-7

**Published:** 2025-10-14

**Authors:** Ivan Vlasov, Tatiana Usenko, Alexandra Panteleeva, Mikhail Nikolaev, Artem Izumchenko, Valeriia Panafidina, Elena Gavrilova, Irina Shlyk, Valentina Miroshnikova, Yurii Polushin, Maria Shadrina, Sofya Pchelina, Petr Slominsky

**Affiliations:** 1 National Research Center «Kurchatov Institute», Moscow, Russian Federation; 2 Pavlov First Saint-Petersburg State Medical University, Saint-Petersburg, Russian Federation; 3 Petersburg Nuclear Physics Institute named by B.P. Konstantinov of National Research Center «Kurchatov Institute», Saint-Petersburg, Russian Federation; 4 Kurchatov Genome Center—PNPI, Saint-Petersburg, Russian Federation

Correction to: *Scientific Reports* 10.1038/s41598-025-00280-3, published online 15 May 2025

The original version of this Article contained an error in the author list, in which the author names were given in the reversed order.

The original Article has been corrected.

