# Supplementary material for: Transcriptomic analysis of differential expression between surviving and nonsurviving patients infected by the SARS-CoV-2 Delta variant
Source: Sci Rep. 2025 May 15;15:16844. doi: 10.1038/s41598-025-00280-3 (PMC12081745; doi:10.1038/s41598-025-00280-3)
Supplement: Supplementary file 1 — Supplementary Material 1 [file 41598_2025_280_MOESM1_ESM.docx]

**Supplementary Table 4 .** Functional cluster selected according to the results of the GO analysis for common DEGs of three pipelines

| GO ID | GO term | padj | Associated Genes, % | Genes |
| --- | --- | --- | --- | --- |
| GO:0006956 | complement activation | 0,0000 | 16,00 | *[C1QA, C1QB, SERPING1, VSIG4]* |
| GO:0050777 | negative regulation of immune response | 0,00000 | 11,11 | *[DHX58, HFE, ISG15, KLRD1, SERPING1, USP18, VSIG4]* |
| GO:0002698 | negative regulation of immune effector process | 0,00112 | 10,26 | *[HFE, KLRD1, SERPING1, VSIG4]* |
| GO:0006959 | humoral immune response | 0,00007 | 8,00 | *[C1QA, C1QB, DEFA3, H2BC4, SERPING1, VSIG4]* |
| GO:0048525 | negative regulation of viral process | 0,00050 | 7,81 | *[IFITM3, ISG15, LY6E, MX1, RSAD2]* |
| GO:0002709 | regulation of T cell mediated immunity | 0,03204 | 6,82 | *[HFE, KLRD1, TRPM4]* |
| GO:0006936 | muscle contraction | 0,00775 | 6,25 | *[JUP, KCNMA1, NMUR1, TRPM4]* |
| GO:0002449 | lymphocyte mediated immunity | 0,00031 | 6,12 | *[C1QA, C1QB, HFE, KIR3DL1, KLRD1, TRPM4]* |
| GO:0002460 | adaptive immune response based on somatic recombination of immune receptors built from immunoglobulin superfamily domains | 0,00244 | 5,62 | *[C1QA, C1QB, HFE, KLRD1, TRPM4]* |
| GO:1903900 | regulation of viral life cycle | 0,00316 | 5,32 | *[IFITM3, ISG15, LY6E, MX1, RSAD2]* |
| GO:0002697 | regulation of immune effector process | 0,00161 | 4,58 | *[EPHB2, HFE, KLRD1, SERPING1, TRPM4, VSIG4]* |
| GO:0051607 | defense response to virus | 0,00052 | 4,40 | *[DHX58, IFI44L, IFI6, IFITM3, ISG15, MX1, RSAD2]* |
| GO:0042742 | defense response to bacterium | 0,03021 | 4,35 | *[DEFA3, H2BC4, ISG15, LYZ]* |
