# Supplementary material for: Transcriptomic analysis of differential expression between surviving and nonsurviving patients infected by the SARS-CoV-2 Delta variant
Source: Sci Rep. 2025 May 15;15:16844. doi: 10.1038/s41598-025-00280-3 (PMC12081745; doi:10.1038/s41598-025-00280-3)
Supplement: Supplementary file 2 — Supplementary Material 2 [file 41598_2025_280_MOESM2_ESM.docx]

**Supplementary Table 3 .** Functional clusters selected according to the results of the GO analysis

| GO ID | GO term | padj | Associated Genes, % | Genes |
| --- | --- | --- | --- | --- |
| **Pipepline 1** | | | | |
| GO:0070106 | interleukin-27-mediated signaling pathway | 0,00001 | 83,33 | *[MX1, OAS1, OAS2, OASL, STAT1]* |
| GO:0071639 | positive regulation of monocyte chemotactic protein-1 production | 0,00081 | 66,67 | *[IL1B, LGALS9, OAS1, OAS3]* |
| GO:0071605 | monocyte chemotactic protein-1 production | 0,00363 | 50,00 | *[IL1B, LGALS9, OAS1, OAS3]* |
| GO:0045071 | negative regulation of viral genome replication | 0,00000 | 35,71 | *[APOBEC3A, EIF2AK2, IFI16, IFIH1, IFIT1, IFIT5, IFITM1, IFITM3, ISG15, MX1, OAS1, OAS3, OASL, RSAD2, TRIM6]* |
| GO:0035456 | response to interferon-beta | 0,00045 | 33,33 | *[IFITM1, IFITM3, OAS1, STAT1, TRIM6, XAF1]* |
| GO:0006120 | mitochondrial electron transport, NADH to ubiquinone | 0,04640 | 28,57 | *[ND3, ND4, ND5, ND6]* |
| GO:0006956 | complement activation | 0,00024 | 28,00 | *[C1QA, C1QB, C2, IL1B, KRT1, SERPING1, VSIG4]* |
| GO:0048525 | negative regulation of viral process | 0,00000 | 26,56 | *[APOBEC3A, EIF2AK2, IFI16, IFIH1, IFIT1, IFIT5, IFITM1, IFITM3, ISG15, LY6E, MX1, OAS1, OAS3, OASL, RSAD2, STAT1, TRIM6]* |
| GO:0071357 | cellular response to type I interferon | 0,00008 | 25,81 | *[IFI27, IFIT1, ISG15, OAS1, OAS3, STAT1, TRIM6, USP18]* |
| GO:0015453 | oxidoreduction-driven active transmembrane transporter activity | 0,01961 | 23,81 | *[COX1, ND3, ND4, ND5, ND6]* |
| **Pipepline 2** | | | | |
| GO:0002227 | innate immune response in mucosa | 0,00087 | 33,33 | *[DEFA3, H2BC11, H2BC21, H2BC4, H2BC7]* |
| GO:0046597 | negative regulation of viral entry into host cell | 0,01108 | 30,77 | *[FCN1, IFITM1, IFITM3, LY6E]* |
| GO:0006956 | complement activation | 0,01311 | 20,00 | *[C1QA, C1QB, FCN1, SERPING1, VSIG4]* |
| GO:0061844 | antimicrobial humoral immune response mediated by antimicrobial peptide | 0,00315 | 19,35 | *[DEFA3, H2BC11, H2BC21, H2BC4, H2BC7, NRG1]* |
| GO:0045071 | negative regulation of viral genome replication | 0,00183 | 16,67 | *[IFIT1, IFITM1, IFITM3, ISG15, MX1, OASL, RSAD2]* |
| GO:0006959 | humoral immune response | 0,00002 | 14,67 | *[C1QA, C1QB, DEFA3, FCN1, H2BC11, H2BC21, H2BC4, H2BC7, NRG1, SERPING1, VSIG4]* |
| GO:0048525 | negative regulation of viral process | 0,00046 | 14,06 | *[FCN1, IFIT1, IFITM1, IFITM3, ISG15, LY6E, MX1, OASL, RSAD2]* |
| GO:0050777 | negative regulation of immune response | 0,02661 | 11,11 | *[DHX58, HFE, ISG15, KLRD1, SERPING1, USP18, VSIG4]* |
| GO:1903900 | regulation of viral life cycle | 0,01076 | 9,57 | *[FCN1, IFIT1, IFITM1, IFITM3, ISG15, LY6E, MX1, OASL, RSAD2]* |
| GO:0045087 | innate immune response | 0,00002 | 7,75 | *[CCL3, DEFA3, DHX58, FCGR3A, FCN1, H2BC11, H2BC21, H2BC4, H2BC7, IFI6, IFIT1, IFITM1, IFITM3, ISG15, KIR3DL1, KLRD1, MX1, SERPING1, USP18, VSIG4]* |
| **Pipepline 3** | | | | |
| GO:0006958 | complement activation, classical pathway | 0,00045 | 75,00 | [C1QA, C1QB, C1QC] |
| GO:0039530 | MDA-5 signaling pathway | 0,00611 | 37,50 | [DHX58, IFIH1, OAS3] |
| GO:0006956 | complement activation | 0,00003 | 24,00 | [C1QA, C1QB, C1QC, C2, SERPING1, VSIG4] |
| GO:0006120 | mitochondrial electron transport, NADH to ubiquinone | 0,03763 | 21,43 | [ND4, ND5, ND6] |
| GO:0060339 | negative regulation of type I interferon-mediated signaling pathway | 0,03763 | 21,43 | [ISG15, OAS3, USP18] |
| GO:0060339 | negative regulation of type I interferon-mediated signaling pathway | 0,03763 | 21,43 | [ISG15, OAS3, USP18] |
| GO:0015453 | oxidoreduction-driven active transmembrane transporter activity | 0,00705 | 19,05 | [COX1, ND4, ND5, ND6] |
| GO:0060337 | type I interferon signaling pathway | 0,02963 | 13,33 | [IFI27, ISG15, OAS3, USP18] |
| GO:0006959 | humoral immune response | 0,00000 | 13,33 | [C1QA, C1QB, C1QC, C2, DEFA3, GNLY, H2BC4, NRG1, SERPING1, VSIG4] |
| GO:0009055 | electron transfer activity | 0,03369 | 12,90 | [COX1, ND4, ND5, ND6] |
