# Supplementary material for: Transcriptomic analysis of differential expression between surviving and nonsurviving patients infected by the SARS-CoV-2 Delta variant
Source: Sci Rep. 2025 May 15;15:16844. doi: 10.1038/s41598-025-00280-3 (PMC12081745; doi:10.1038/s41598-025-00280-3)
Supplement: Supplementary file 3 — Supplementary Material 3 [file 41598_2025_280_MOESM3_ESM.docx]

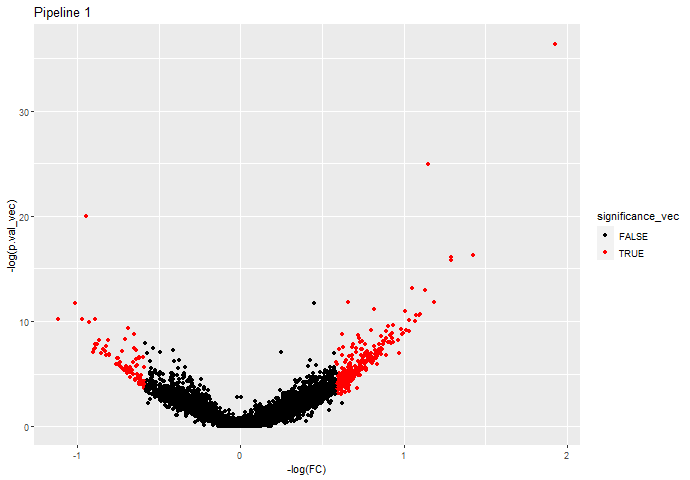

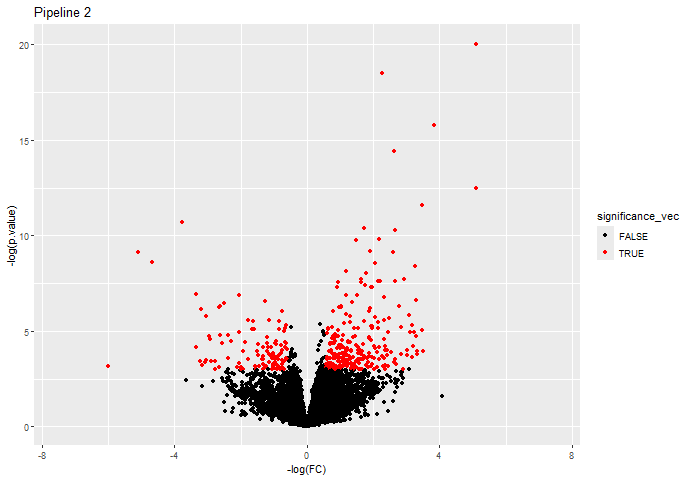

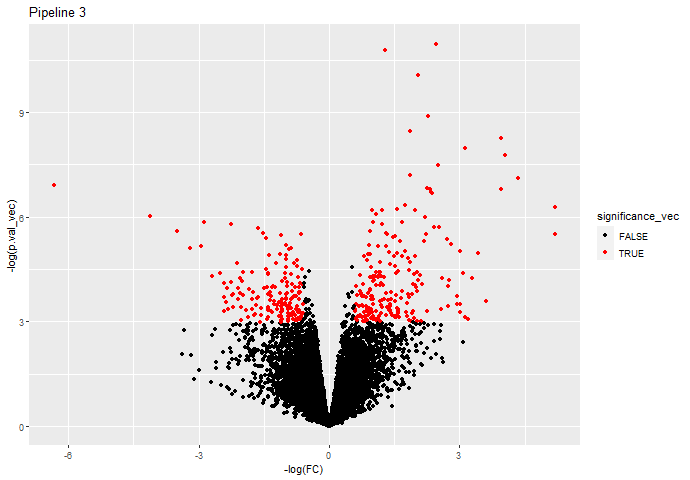


Supplementary figure 1. Volcano plots (-log of fold change/- log of p.value) of all genes for each pipeline.
